# Impact of removable complete dentures on quality of life and social dimension: a cross-sectional patient-reported study

**DOI:** 10.3389/fdmed.2026.1883309

**Published:** 2026-07-02

**Authors:** Saverio Ceraulo, Antonio Barbarisi, Erda Qorri, Dorina Lauritano, Gianluigi Caccianiga, Francesco Carinci

**Affiliations:** 1 School of Medicine and Surgery, University of Milano-Bicocca, Monza, Italy; 2 Dental School, Department of Medicine and Surgery, University of Milano-Bicocca, Milan, Italy; 3 Dental School, Albanian University, Tiranë, Albania; 4 Department of Translational Medicine and for Romagna, University of Ferrara, Ferrara, Italy

**Keywords:** complete dentures, edentulism, OHRQoL, prosthesis rehabilitation, social impact

## Abstract

Total tooth loss significantly impacts chewing, phonation, and facial aesthetics, negatively impacting overall well-being.

**Background/objectives:**

This study aims to evaluate patients' perceptions of complete removable dentures in relation to their quality of life, with particular attention to functional, psychological, and social dimensions.

**Methods:**

A cross-sectional observational study was conducted to analyze the subjective perceptions of patients wearing complete removable dentures. Fifty completely edentulous patients were selected, 27 men and 23 women, aged between 65 and 83 years. A structured 10-item questionnaire was developed by selecting and adapting items from the OHIP-EDENT framework, focusing on three dimensions: functional comfort, psychological well-being, and social participation. This instrument represents neither the original OHIP-EDENT nor a formally validated translation or cross-cultural adaptation; it should be considered an exploratory measure of patient-reported outcomes, and its unvalidated nature is an acknowledged methodological limitation of this study. Responses were recorded using a 5-point Likert scale. The questionnaire's internal consistency was assessed using Cronbach's Alpha, Given the ordinal nature of the Likert scale data, the Friedman test was used as the primary inferential statistical method to compare differences between the three dimensions, with one-way repeated measures ANOVA applied as a complementary parametric analysis (*p* < 0.05).

**Results:**

The mean scores showed higher values in the functional dimension (3.37 ± 1.18) and in the psychological dimension (3.23 ± 1.05), while the social dimension showed a significantly lower score (2.06 ± 1.01), indicating greater difficulties in social relationships. The repeated measures analysis of variance confirmed that these differences are not random, but statistically significant with results of F(2,98) = 52.4 *p* < 0.001. The Friedman test further confirmed these differences X2(2) = 48, *p* < 0.001 strengthening the solidity of the results.

**Conclusion:**

Within the limits of this cross-sectional observational study, and without any claim to causal inference, the social dimension showed the lowest scores reported by patients in this sample, suggesting it may represent an area of greater perceived difficulty. These results are exploratory and descriptive and cannot be interpreted as evidence of a direct effect of prosthetic rehabilitation on quality of life.

## 1 Introduction

Complete edentulism remains a widespread clinical condition, particularly among the elderly population, and constitutes a significant problem not only from a functional perspective, but also from a psychological and social one. The complete loss of teeth has significant repercussions on chewing ability, phonation, and facial aesthetics, with significant impacts on the individual's overall well-being (1, 2). In recent years, research attention has progressively shifted toward a broader, patient-centered evaluation of treatment outcomes, including not only objective clinical parameters but also the patient's subjective perception and level of satisfaction (3). Rehabilitation with removable complete dentures continues to represent one of the main therapeutic options for the treatment of complete edentulism, especially due to its accessibility and relative ease of fabrication compared to implant-based dentures (4, 5). Treatment success cannot be defined solely in terms of the stability or functionality of the prosthesis, but must necessarily include the impact on the patient's quality of life. The concept of oral health-related quality of life (OHRQoL) has assumed a central role in contemporary dental research (6). Numerous studies, including recent systematic reviews and meta-analyses, have consistently shown that edentulous patients report a significantly lower quality of life than individuals with natural teeth. This association has been confirmed in different populations and with various measurement tools, such as assessments based on the OHIP and GOHAI, and appears to influence both oral health and general well-being. Specifically, tooth loss and edentulism have been linked not only to functional limitations, such as reduced chewing efficiency and dietary restrictions, but also to psychosocial impacts, including lower self-esteem and social discomfort in daily interactions. Evidence from a recent systematic review and meta-analysis further strengthens this relationship, showing a strong association between increasing tooth loss and worsening quality of life. Similarly, clinical evidence from periodontitis patients indicates that edentulism is associated with significantly impaired quality of life, while prosthetic rehabilitation has been associated with better outcomes in both functional and psychosocial dimensions (7–9). Beyond the direct improvement in oral function and patient-reported quality of life, complete denture rehabilitation may also influence broader aspects of health, particularly in older adults. Improved chewing function has been associated with the consumption of a wider variety of foods, potentially contributing to improved nutritional status and reducing some of the dietary limitations commonly observed in edentulous individuals. This aspect is particularly relevant in frail and medically compromised patients, in whom impaired chewing ability has been associated with poorer nutritional intake and reduced functional capacity. Recent evidence suggests that complete denture wearers may report differences not only in oral function but also in general well-being, social participation, and everyday functioning. However, these outcomes are not uniform across all patients. Factors such as frailty status, nutritional condition, adaptation to the prosthesis, previous denture experience, residual oral anatomy, and individual expectations appear to play a significant role in determining treatment success. Consequently, while complete dentures generally provide substantial benefits for edentulous individuals, the reported outcomes vary considerably according to the patient's clinical and systemic characteristics (10–13). Patient satisfaction is closely related not only to the technical quality of the prosthesis, but also to individual expectations, personality and previous experience; these aspects determine the level of perceived well-being (14). This reinforces the importance of a holistic approach in the evaluation of patients wearing complete dentures. To systematically assess the impact of oral health on quality of life, standardized and validated tools have been developed, including the Oral Health Impact Profile (OHIP) and its variants. These tools allow a reliable quantification of functional limitations, psychological distress and social difficulties associated with oral conditions (15). The use of patient-reported outcome measures (PROMs) represents a fundamental approach in evaluating the effectiveness of a given treatment and for optimizing therapeutic interventions, taking into account the patient's point of view, which is essential for evaluating clinical efficacy and perceived quality of life (16). The relationship between functional improvement and subjective perception is not always linear, and a clinically adequate denture does not necessarily translate into a high level of patient satisfaction. Despite the growing number of studies on oral health-related quality of life (OHRQoL) in edentulous populations, the functional, psychological, and social dimensions have rarely been examined as distinct outcomes within the same cohort of patients fitted with conventional complete removable dentures. In particular, the relative weight of the social dimension—which includes interpersonal comfort, self-confidence in public, and participation in shared daily activities—remains inadequately characterized in this clinical population. This study fills this gap by evaluating patients' subjective perceptions of comfort, psychological well-being, and social participation in edentulous elderly patients rehabilitated with complete removable dentures, with the aim of generating clinically relevant evidence to support a more holistic approach to prosthetic rehabilitation.

## 2 Materials and methods

**Figure 1:**
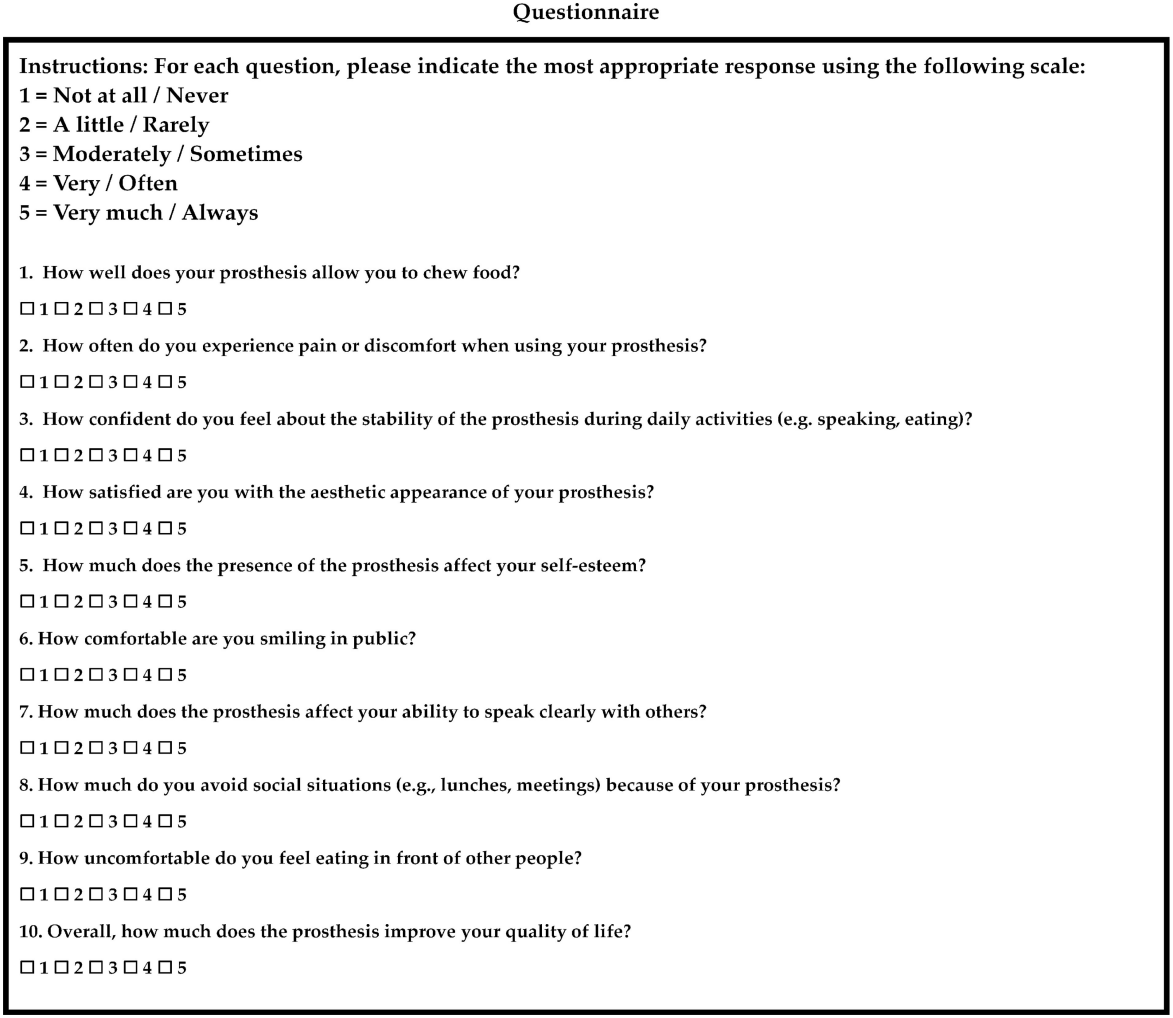
Questionnaire administered to patients.

A cross-sectional observational study was conducted to analyze the subjective perception of patients with removable complete dentures, with particular attention to aspects related to quality of life and social issues. This study was chosen to obtain a realistic snapshot of patients' experiences at a specific point in time, without affecting clinical variables. The study was conducted in accordance with the Declaration of Helsinki and national ethical guidelines for research on humans. The study protocol was approved by the Albanian University Ethics Committee (n°9 prot dt 09.01.2024). Participation was spontaneous and voluntary, and each participant provided written informed consent, with the option to withdraw at any time. All anonymous responses were collected securely, ensuring full confidentiality and protection of participants' personal data. For the study, 50 totally edentulous patients were selected, 27 men and 23 women, aged between 65 and 83 years, attending a dental practice. Patient recruitment was based on voluntary participation. A total of 62 completely edentulous patients attending the dental practice were invited to participate in the study; of these, 50 agreed to take part and provided written informed consent, resulting in a participation rate of approximately 80.6%. The remaining 12 patients declined to participate for personal reasons or did not meet the inclusion criteria. No formal *a priori* sample size calculation was performed, as recruitment followed a consecutive convenience sampling approach. All participants were recruited from a single private dental practice located in Tirana, Albania. This single-center recruitment approach, while limiting the generalizability of the results, contributed to the relative homogeneity of the sample, as all patients shared a comparable clinical background, similar access to private dental care, and a substantially uniform socioeconomic and cultural background. Inclusion criteria were: having worn a removable mono- or bimaxillary complete denture for at least 12 months, to ensure adequate adaptation to the denture and minimize the impact of any initial adjustment difficulties on subjective perception; absence of uncompensated systemic diseases that could affect general well-being or cognitive function, including uncontrolled diabetes mellitus or clinically significant cardiovascular disease, absence of diagnosed psychiatric disorders, adequate understanding of the research project and the administered questionnaire, verified by the dentist prior to enrollment, provision of written informed consent; and absence of active oral or systemic infections at the time of enrollment. Exclusion criteria were: presence of cognitive impairment sufficient to impair understanding of the questionnaire or research procedures, assessed by clinical observation and confirmed by the treating dentist, diagnosis of psychiatric or psychological disorders that could significantly alter the subjective perception of oral health-related quality of life, inability to understand the aims and procedures of the research project, refusal to sign the informed consent form, presence of active infections at the time of enrollment. All patients who signed the informed consent form were administered an anonymous structured questionnaire derived from the OHIP-EDENT framework. The instrument used in this study was neither the original OHIP-EDENT questionnaire nor a formally validated cross-cultural adaptation. Instead, a 10-item questionnaire was independently developed, selecting and adapting items from the OHIP-EDENT framework deemed clinically relevant for the three dimensions investigated: functional comfort, psychological well-being, and social participation. No formal psychometric validation was conducted prior to administration. The questionnaire was administered in Albanian by a trained research assistant and was intended solely as an exploratory measure of patient-reported outcomes. The use of a non-validated instrument is an acknowledged limitation of this study, explicitly noted in the Limitations section. Comparisons with studies that used the original or formally validated OHIP-EDENT should therefore be made with caution. The responses were recorded using a 5-point Likert scale (1 = Not at all/Never; 2 = A little/Rarely; 3 = Moderately/Sometimes; 4 = Very/Often; 5 = Very much/Always) (Figure 1). Any doubts regarding the understanding of the questions were clarified with direct assistance, without influencing the answers. It was completed in a quiet home environment to promote concentration and reduce potential bias. The internal consistency of the questionnaire was assessed using Cronbach's Alpha, with values ≥ 0.7 considered indicative of good reliability, thus confirming the instrument's reliability. Given the ordinal nature of the Likert scale data and the non-normal distribution of scores—as confirmed by the Shapiro–Wilk test applied before the main analyses—the Friedman test was selected as the primary inferential statistical method to compare differences between the three dimensions (functional, psychological, and social), with *post-hoc* pairwise comparisons performed using the Wilcoxon signed-rank test with Bonferroni correction. A one-way repeated-measures ANOVA was additionally applied as a supplementary parametric analysis, in line with approaches reported in comparable published studies on oral health-related quality of life. Given the ordinal nature of Likert scale data, this parametric analysis must be regarded as secondary and its results interpreted with appropriate caution. The convergence of findings from both the non-parametric Friedman test and the supplementary ANOVA was considered supportive of the consistency of the observed pattern of results, without implying that parametric assumptions were fully satisfied. All tests were considered statistically significant at *p* < 0.05. The collected data were encoded in Excel format and imported into Jamovi version 2.3 (free and open-source statistical software) for analysis, ensuring the transparency and replicability of the calculations. Despite a sample of 50 participants, the study's size allows for a preliminary analysis of the differences between the functional, psychological, and social dimensions of participants' quality of life.

## 3 Results

**Figure 2:**
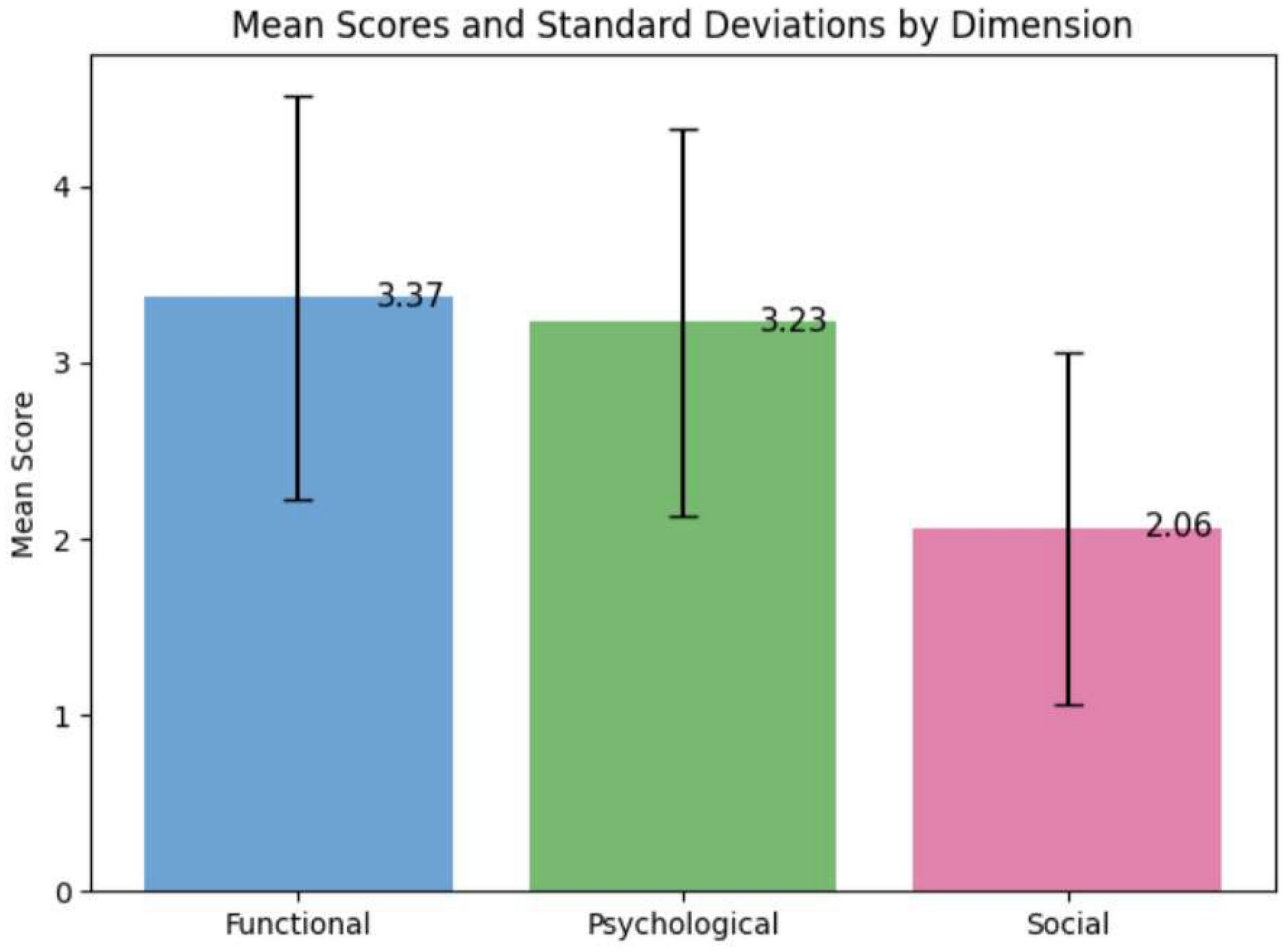
Means and standard deviations of scores in the three dimensions of the OHIP-EDENT questionnaire (functional, psychological and social) in patients wearing removable complete dentures.

Data from 50 patients were analyzed with prior written consent; all patients completed the questionnaire. Given the ordinal nature of the Likert response scale and the relatively modest sample size (*n* = 50), the assumption of a normal distribution cannot be considered reliable for this type of data. For this reason, the Friedman test was chosen as the primary inferential statistical method, as it does not require distributional assumptions and is specifically suited to ordinal repeated measures. A one-way ANOVA for repeated measures was also performed as a complementary analysis, in line with approaches used in comparable studies on oral health-related quality of life, to verify whether the pattern of results held even under parametric assumptions. The fact that both methods converged on the same statistically significant results was considered further confirmation of the robustness of the results. Mean scores showed higher values in the functional (3.37 ± 1.18) and psychological (3.23 ± 1.05) dimensions, while the social dimension showed a significantly lower score (2.06 ± 1.01), indicating greater difficulties in social relationships (Figure 2). The additional repeated measures ANOVA also confirmed statistically significant differences [F(2,98) = 52.4, *p* < 0.001]. Given the cross-sectional design and the exploratory nature of the instrument, these results should be interpreted as descriptive: they indicate an association within this sample, not a causal effect of prosthetic rehabilitation. Internal reliability using Cronbach's alpha showed a value of 0.78, indicating good overall consistency of the questionnaire; this suggests that the questionnaire questions, despite exploring different aspects, are sufficiently consistent with each other in measuring the impact of prostheses on quality of life. In addition, the Friedman test was performed which showed that the differences between the three dimensions were statistically significant X2(2) = 48, *p* < 0.001 strengthening the robustness of the results obtained with the ANOVA. Overall, the results indicate that, despite generally satisfactory prosthetic function and reasonable psychological adjustment, significant critical issues remain in the social sphere, which is the area most affected by prosthetic use. The *p* values <0.001 for both tests confirm that the observed differences between the three dimensions are statistically significant. The ANOVA result, being supplementary, should however be interpreted with caution given the ordinal nature of the data (Table 1).

**Table 1:** The ANOVA and friedman test values refer to the overall comparison between the three dimensions. The Cronbach's *α* value (0.78) reflects the internal consistency of the complete 10-item questionnaire.

Dimension	Media ± SD	Cronbach's *α*	One-way ANOVA F (df = 2.98)	*p*-value (ANOVA)	Test Friedman X^2^ (df = 2)	*p*-value (Friedman)
Functional	3.37 ± 1.18	0.78	52.4	<0.001	48.3	<0.001
Psychological	3.23 ± 1.05	0.78	—	—	—	—
Social	2.06 ± 1.01	0.78	—	—	—	—

## 4 Discussion

**Figure 3:**
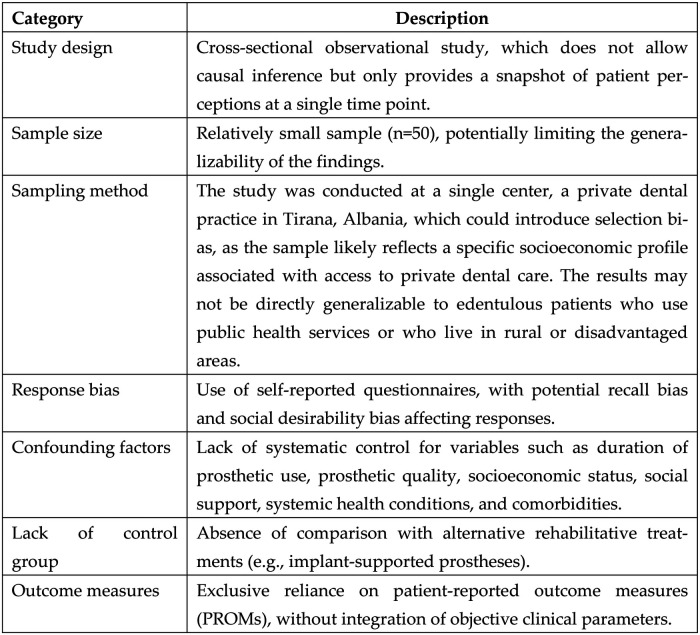
Bias and limitations of the study.

The results of the present study show that, although patients wearing complete removable dentures report relatively high levels of satisfaction in the functional and psychological dimensions of oral health-related quality of life, the social dimension is significantly different, with a significantly lower mean score (2.06 ± 1.01) compared to the other two dimensions. This pattern is not isolated, but reflects a dynamic already observed in the edentulous population rehabilitated with complete removable dentures: previous studies have reported favorable functional outcomes in complete denture wearers, but the subjective perception of one's social role and daily interaction with others requires a more complex and prolonged adaptation over time (17). Recent studies have reported higher OHRQoL scores among complete denture wearers up to one year after treatment, particularly for domains related to chewing ability and pain perception, in particular for the components related to chewing and pain reduction, but the effects on social and psychological perceptions are more variable and influenced by contextual and individual factors as well as environmental ones (18). The study population, although small, was recruited exclusively from a single private dental practice in Tirana, Albania. This characteristic contributed to a certain degree of clinical and contextual homogeneity among participants, given their shared access to private dental care and comparable socioeconomic and cultural backgrounds. It should be noted, however, that this very characteristic limits the external validity of the results, as the sample may not be fully representative of the broader edentulous elderly population, including individuals attending public health facilities or living in rural areas. The social dimension includes aspects deeply rooted in self-perception, personal confidence and interpersonal relationships. Some patients reported difficulties in dealing with social situations such as group conversations, smiling in public or eating meals with others without fear of judgement, and these difficulties are not always overcome by the simple presence of an adequate prosthesis. In a longitudinal study, the overall OHRQoL of edentulous patients improved more with implant-supported prostheses than with conventional prostheses, especially for comfort, chewing and perception of aesthetics, highlighting how greater prosthetic stability was associated with higher levels of perceived social trust and participation (19, 20). These findings further support the importance of considering not only clinical, but also psychological and social relational factors in the rehabilitation process. The literature reports that structural determinants of health contribute to a good quality of life related to oral health (21). Patients who are more socially satisfied tend to have realistic expectations, adequate family support and active social networks. Prosthetic rehabilitation thus becomes a deeply personal and sensorial experience, linked to how the individual perceives himself in his daily life context. The present findings suggest that, even with a well-constructed prosthesis, concerns remain related to aesthetic acceptance, denture stability and fear of embarrassment in a social context, elements that can limit the perceived effectiveness of the treatment (22, 23). The non-parametric Friedman test confirms that the social dimension represents the area of greatest perceived difficulty in this sample, in line with what is reported in the literature for this patient population. The results of this study, although based on a medium-sized sample, are consistent with studies present in the literature that underline the importance of a holistic approach in the care of patients wearing complete dentures (24, 25). They call for the development of clinical protocols that are not limited to the construction of an adequate prosthesis, but also include psychological assessments, educational programs for prosthesis adaptation and follow-up strategies that promote social participation and overall well-being of patients (26). Despite the clinical interest of these findings, several limitations must be acknowledged (Figure 3): the cross-sectional design prevents causal inference; the sample is small (*n* = 50) and recruited from a single private center, which limits generalizability; no control group was included; the study relied solely on PROMs without adjusting for confounders such as prosthesis duration, prosthesis quality, socioeconomic status, social support, and systemic comorbidities; and the instrument, although showing acceptable internal consistency (Cronbach's *α* = 0.78), was neither the original OHIP-EDENT nor a formally validated adaptation, so comparisons with studies using the validated version should be made with caution. The decision not to include a control group of edentulous patients who had not been rehabilitated or treated with alternative solutions was motivated by ethical and practical considerations. Specifically, recruiting completely edentulous subjects without prosthetic rehabilitation could expose patients to significant functional and social distress, contradicting the principles of patient-centered care and respect for individual well-being. Furthermore, the primary focus of the study was to assess the subjective perception of patients already adapted to complete removable dentures, in order to understand differences between functional, psychological, and social dimensions, rather than comparing treatment modalities. We acknowledge that the lack of a control group limits the possibility of direct comparisons between different treatment modalities. However, it is important to emphasize that the primary objective of this study was never to compare different rehabilitation approaches, but rather to explore, from the patient's perspective, how the three dimensions of quality of life—functional, psychological, and social—are experienced by individuals who have been using complete removable dentures for at least a year. In this sense, the internal analysis of differences between the three dimensions provides descriptive and exploratory data on patients' self-reported perceptions at a specific and clinically significant point in their rehabilitation journey. The study's cross-sectional design and the lack of a pre-treatment baseline assessment preclude any causal inferences. The observed gap between the social dimension and the other two domains, however, is of clinical interest, as it suggests that functional restoration alone may not be sufficient to meet the full spectrum of patients' needs (Figure 3).

## 5 Conclusions

Within the limits of this cross-sectional observational study, differences in patient-reported scores were observed across the functional, psychological, and social dimensions, suggesting that prosthetic rehabilitation should be approached holistically, paying attention to psychological and social well-being in addition to technical function. No causal inferences can be drawn from these results. Both the Friedman test (*p* < 0.001) and the complementary repeated-measures ANOVA (*p* < 0.001) identified statistically significant differences among the three investigated dimensions. Within this sample, the social dimension showed lower scores than the functional and psychological dimensions, suggesting that social aspects may represent an area of greater perceived difficulty among complete denture wearers. Future studies should include larger multicenter samples, pre- and post-treatment assessments, formally validated instruments, and appropriate comparison groups to test whether the pattern observed here is reproducible and to identify the factors that most influence social well-being in this patient population. It should be noted that, given the cross-sectional nature of this study, the results reflect patients' subjective perceptions at a single point in time and should not be interpreted as evidence of a causal relationship between prosthetic rehabilitation and quality of life.

## 6 Clinical significance

In this sample, participants reported higher scores on the functional and psychological dimensions than on the social dimension, suggesting that complete removable dentures may promote functional recovery but do not automatically restore confidence in social interactions. Clinicians should consider integrating social well-being assessments into routine care, alongside functional and aesthetic assessments, and explore supportive strategies such as patient education and structured follow-up aimed at social participation. These recommendations are preliminary, based on a single small cross-sectional study using a non-validated instrument.

## Institutional review board statement

This study was conducted in accordance with the Declaration of Helsinki, Albanian University Ethics Committee (n°9 prot dt 09.01.2024).

## Data Availability

The original contributions presented in the study are included in the article/Supplementary Material, further inquiries can be directed to the corresponding author.
